# Supplementary figures and images for: Treatment pathways traversed by polycystic ovary syndrome (PCOS) patients: A mixed-method study
Source: PLoS One. 2021 Aug 9;16(8):e0255830. doi: 10.1371/journal.pone.0255830 (PMC8351971; doi:10.1371/journal.pone.0255830)

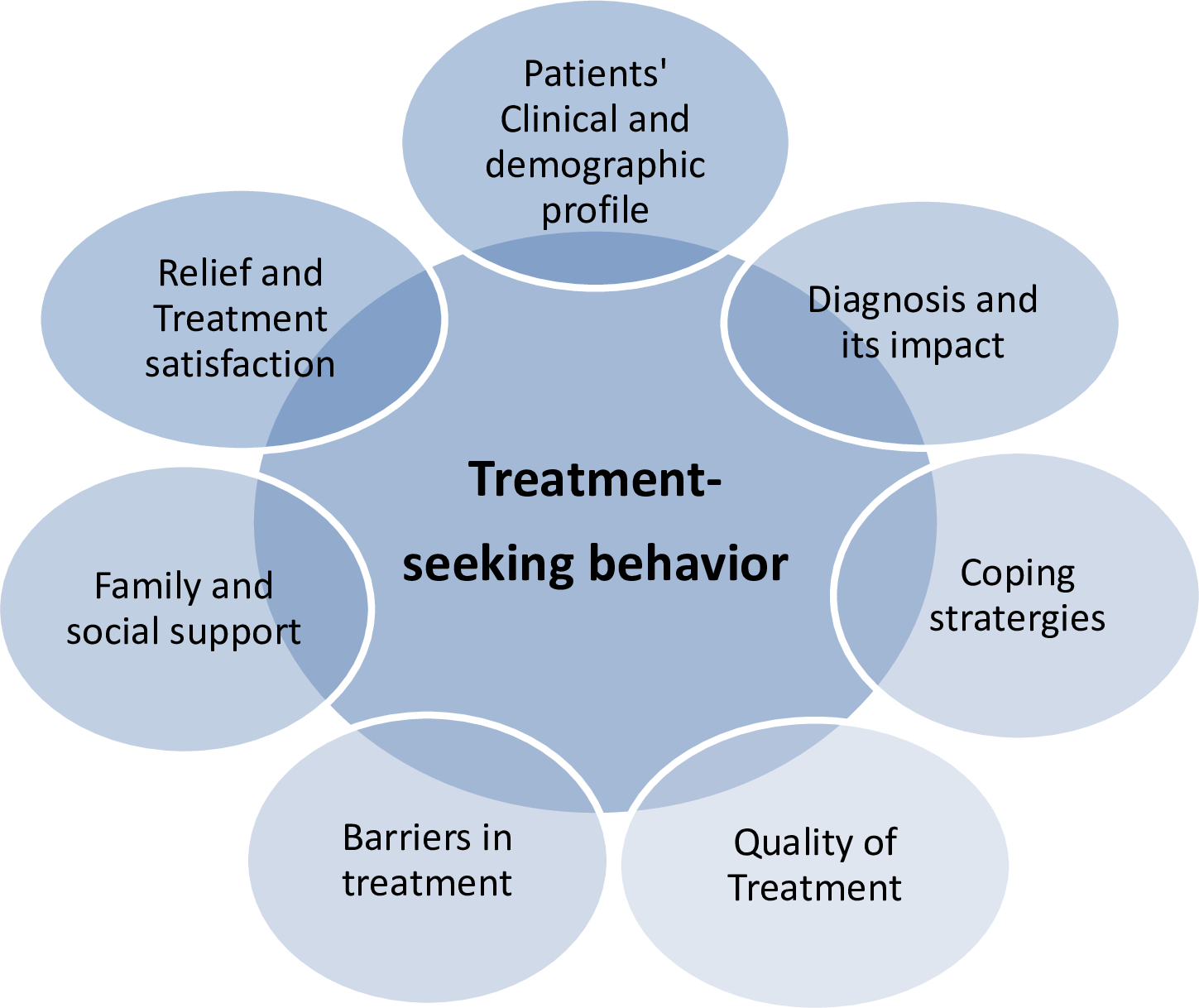

Supplement: S1 Fig — It was developed from a literature review of the determinants affecting treatment-seeking behavior. (TIF) [file pone.0255830.s002.tif]
