# Supplementary material for: Treatment pathways traversed by polycystic ovary syndrome (PCOS) patients: A mixed-method study
Source: PLoS One. 2021 Aug 9;16(8):e0255830. doi: 10.1371/journal.pone.0255830 (PMC8351971; doi:10.1371/journal.pone.0255830)
Supplement: Attachment — Submitted filename: Doc2.edited.docx [file pone.0255830.s004.docx]

**Treatment pathways traversed by polycystic ovary syndrome (PCOS) patients: A mixed**

**method study.**

This is an exciting piece of research addressing the awareness, information source and coping strategies in women with PCOS.

**Comment:** the abstract section of the study is too long. The method section should summarise concisely the method used. Similarly, the result section of the abstract should only summarise the main results. The conclusion should be more focused and reflect the results of the study. Overall, the abstract should be flawless for the readers, not too extended and more focused.

**Comment:** Financial disclosure, please provide grant number or reference.

In the introduction, the author claimed that “Polycystic ovary syndrome (PCOS), a gynaecological morbidity". PCOS is an endocrine condition affecting women of reproductive age; the primary pathology is hormonal disturbances which drives to metabolic, gynaecological and reproductive consequences.

**Comment:** in the introduction, the author should refer to the recent "international evidence-based guideline for assessing and managing polycystic ovary syndrome 2018". Which highlighted the importance of evaluating the QoL aspect of PCOS.

**Comment:** the study was designed as a mixed-method triangulation; this method is suitable when combining quantitative and qualitative methods to answer a specific research question. This method leads to a better explanation and link different aspects of a single research question.

**Comment:** in the method section, please move reference 16 to Rotterdam criteria instead of the end of the text.

**Comment:** Data collection: this section should be the intervention section which will reflect the questionnaire administered in the study as the author was used an Excel spreadsheet to collect data. For the quantitative data, it is not clear whether the questionnaire used was a validated questionnaire? What was the scale used to capture responses, for instance, the 7-point-Likert scale?. The author has piloted the questionnaire with eight patients; what were the results? Whether any modification to the questionnaire was made as a result of piloting?

**Comment:** COREQ guidelines, please use full text (no abbreviation) when it first used.

**Comment:** The patient's responses were captured in another language which later translated into English. Does this create any risk of bias for the accurateness of the translation? Did the author use approved translation software or translator? It isn't easy to translate word to word from any other language to English.

**Comment:** The technical details should be expanded and clarified to ensure that readers understand exactly what the researchers studied.

**Comment:** the statistical analysis session is too long; this should be short and concise.

**Comment:** the author mentioned, "This study objective was an ancillary part of a larger PhD project entitled “Efficacy of probiotic-based dietary and lifestyle regime in the management of PCOS cases", this should be removed from the method section of the manuscript and should be in the conflict of interest section.

**Comment:** the manuscript lacks clear inclusion/exclusion criteria for the participants.

**Comment:** in the quantitative section of the results, please give exact numbers instead of writing “remaining”.

**Comment:** what is “viz” mentioned in the manuscript?

**Comment:** table 2 is complicated to follow the trends, very complicated for readers to understand precisely what is happening. It should be simplified further.

**Comment:** overall, the results section is very long. This should be more concise and focused.

**Comment:** the results and the discussion should be separate sections.

**Comment:** the discussion section is very repetitive. Please rewrite this section and make it more concise.

**Comment:** Line 447, **Figure 1: Pathways to treatment traversed by PCOS patients.** This should be removed from the discussion and only refer to the figure within the text, for example (Figure 1). Please remove any bullet points from the text. This section should be in the result section.

**Comment:** the author claimed, “Five major factors emerged from this data, i.e."; this should be in the result section instead of the discussion.

**Comment:** one of the limitations of this study is that the questionnaire was administered in a language other than English, then translated to English. This could lead to a risk of bias in interpreting the script word to word.

**Comment:** the conclusion section is very poor; hardly any main results were mentioned. Additionally, this section is very long; it must be short, concise and summarising the main results of the study.

**Comment:** reference 1, 23, 25, 26,29,32, 33,34, please add DOI.

**Comment**: overall, the language is unclear, making it difficult to follow. I advise the authors work with a writing coach or copyeditor to improve the flow and readability of the text.

**Comment:** what this study will add to the field?

**Comment:** reading this study, again and again, I do not know what precisely the main results are. This should be very clear and consistent throughout the text, from the abstract to the conclusion.
